# Reciprocal Serum Phosphatidylcholine Signatures Are Related to Intestinal Inflammation in Inflammatory Bowel Disease and Liver Fibrosis in Primary Sclerosing Cholangitis—An Exploratory Study

**DOI:** 10.3390/biomedicines14071485

**Published:** 2026-06-30

**Authors:** Tanja Elger, Muriel Huss, Hauke Christian Tews, Marcus Höring, Johanna Loibl, Arne Kandulski, Martina Müller, Gerhard Liebisch, Christa Buechler

**Affiliations:** 1 Department of Internal Medicine I, Gastroenterology, Hepatology, Endocrinology, Rheumatology, and Infectious Diseases, University Hospital Regensburg, 93053 Regensburg, Germany; tanja.elger@klinik.uni-regensburg.de (T.E.); muriel.huss@klinik.uni-regensburg.de (M.H.); hauke.tews@klinik.uni-regensburg.de (H.C.T.); johanna.loibl@klinik.uni-regensburg.de (J.L.); arne.kandulski@klinik.uni-regensburg.de (A.K.); martina.mueller-schilling@klinik.uni-regensburg.de (M.M.); 2 Institute of Clinical Chemistry and Laboratory Medicine, University Hospital Regensburg, 93053 Regensburg, Germany; marcus.hoering@klinik.uni-regensburg.de (M.H.); gerhard.liebisch@klinik.uni-regensburg.de (G.L.)

**Keywords:** phosphatidylcholine, inflammatory bowel disease, liver fibrosis, primary sclerosing cholangitis, fecal calprotectin, C-reactive protein

## Abstract

**Background:** Phosphatidylcholine (PC) is a major phospholipid that contributes to intestinal barrier protection and is essential for hepatic secretion of lipids and bile acids. Because inflammatory bowel disease (IBD) and primary sclerosing cholangitis (PSC) are closely linked, we hypothesized that individual serum PC species would reflect disease activity. We therefore investigated whether serum PC profiling could identify clinically useful biomarkers across the gut–liver axis. **Methods:** Serum concentrations of 21 PC species were quantified by direct flow injection high-resolution mass spectrometry in 16 healthy controls, 57 patients with IBD, and 20 patients with PSC. **Results:** In IBD, multiple serum PC species were inversely associated with inflammatory activity, showing negative correlations with serum C-reactive protein and fecal calprotectin. Patients with fecal calprotectin concentrations above the diagnostic cut-off of 120 µg/g had lower levels of PC 34:3, 36:1, 36:2, 36:3, 36:4, 36:5, 38:3, 38:4, 38:5, 38:7, 40:5, and 40:6, as well as lower total PC. In contrast, in PSC, PC 30:0, 32:0, 32:1, and 34:1 were increased compared with IBD and correlated positively with gamma-glutamyltransferase and alkaline phosphatase. Furthermore, these shorter-chain PC species as well as PC 36:1 were markedly elevated in PSC with advanced liver fibrosis compared with PSC without fibrosis. **Conclusions:** Serum PC species show a reciprocal disease-associated pattern in IBD and PSC. In IBD, lower concentrations of predominantly unsaturated PC species are associated with active intestinal inflammation, whereas in PSC, higher concentrations of shorter-chain PC species are associated with cholestatic injury and advanced liver fibrosis. IBD and PSC exhibit opposing serum PC signatures, suggesting that dysregulated PC metabolism is a pathophysiological feature of intestinal inflammation and PSC-associated liver fibrosis.

## 1. Introduction

Crohn’s disease (CD) and ulcerative colitis (UC) are the primary types of inflammatory bowel disease (IBD), a group of conditions characterized by persistent, recurrent inflammation of the gastrointestinal tract. While the precise cause of IBD is not fully understood, substantial evidence indicates that a combination of genetic predisposition and environmental influences plays a significant role in determining an individual’s risk of developing the disorder [1,2,3].

Phosphatidylcholine (PC) is a phospholipid well known for its role in lipid homeostasis and bile acid excretion [4,5]. In bile fluid, PC protects epithelial cells from the cytotoxic effects of bile acids [6]. Notably, there is also evidence that PC within the intestinal mucus layer prevents bacterial invasion and subsequent inflammation [7].

In patients with chronic inflammatory and autoimmune diseases, alterations in serum PC species profiles have been reported. For instance, patients with rheumatoid arthritis exhibit higher levels of saturated PC species and lower levels of PC species containing the essential fatty acids linoleic and linolenic acids [8]. A decline in serum PC 36:4, 38:4, and 38:5 levels has been observed in patients with polymyositis or dermatomyositis compared with healthy controls [9].

Several studies have investigated PC levels in the circulation of patients with IBD. However, Fan et al. reported normal plasma PC concentrations in patients with UC and CD [10]. In contrast, other studies found decreased plasma PC levels in patients with CD compared with healthy controls, whereas reductions in UC were not statistically significant [11,12]. A limitation of these studies is the small cohort size, with 18 or 40 patients included [10,11,12], which may prevent the identification of significant differences. Moreover, whereas C-reactive protein was normal in the study by Fan et al., patients’ C-reactive protein levels were elevated in the study by Iwatani et al. [10,12].

Because total PC levels were also reduced in patients with IBD and severe inflammation compared with those with inactive disease [13], differences in inflammatory status may explain the discordant findings across studies [10,11,12]. Moreover, it has been shown that patients with IBD who responded to anti-TNF therapy exhibited lower serum PC levels but markedly increased fecal PC levels compared with controls. As the difference in serum PC levels was small, this result needs confirmation [14]. Taken together, these findings do not clarify whether plasma PC levels decline in IBD or whether they are related to disease activity. A bidirectional Mendelian randomization study demonstrated that the PC species PC 16:0/20:4 (the main species of PC 36:4 [15]), PC 16:0/20:5, and PC 18:0/20:5 are protective in UC, CD, and IBD in general [16], suggesting that reduced levels of polyunsaturated PC species may contribute to IBD pathogenesis.

IBD is closely associated with primary sclerosing cholangitis (PSC), a progressive inflammatory disease of the bile ducts leading to liver fibrosis and finally resulting in liver cirrhosis. Currently, there is no curative treatment available besides orthotopic liver transplantation [17,18,19]. Stremmel et al. hypothesized that impaired paracellular transport of PC to the apical side of cholangiocytes leads to depletion of PC in the biliary mucus, potentially contributing to PSC pathogenesis [20]. PC in the apically located mucus of the biliary epithelium appears to originate from the circulation by passing through the tight junction barrier [20]. Notably, biliary PC levels in patients with PSC have been reported to be normal [21]. However, total bile acid levels and choline-containing phospholipids in the gallbladder were also described to be lower in PSC patients than in healthy controls [22]. These studies included very few patients, as PSC is a rare disease, and did not differentiate between PSC-IBD and PSC [21,22]. Thus, whether disturbed biliary PC levels have a role in PSC/PSC-IBD requires further study.

To date, whether serum PC levels or the composition of PC species in the serum of PSC patients is altered has not been evaluated. Moreover, the relationship between serum PC species and chronic liver injury in patients with PSC remains unresolved.

In individuals with non-alcoholic fatty liver disease, serum PC 16:1/22:6 levels were inversely correlated with the proinflammatory cytokines interleukin (IL)-1β and tumor necrosis factor (TNF), suggesting that this species declines in these patients [23], consistent with previous findings of reduced plasma PC levels [24]. In patients with liver cirrhosis, polyunsaturated PC species generally decreased, whereas saturated PC species were increased [25,26]. Serum PC 32:0 was identified as a marker of liver cirrhosis in patients with hepatitis C virus (HCV) infection, both before and after antiviral therapy [25]. Other studies, however, found no association between systemic PC levels and liver cirrhosis. Meikle et al. compared 31 patients with alcoholic cirrhosis to 28 chronic drinkers without liver disease and did not account for species-specific effects. Virseda-Berdices et al. included patients with HIV/HCV coinfection, who may differ from non-infected controls [27,28].

Despite clear evidence that PC is critical for intestinal, biliary, and hepatic function—and that these roles depend on the specific composition of PC species—serum levels of PC species, to our knowledge, have not been directly compared between patients with IBD and those with PSC.

Data on individual serum PC species in IBD are scarce, and such analyses are lacking in PSC. We hypothesized that active IBD is associated with reduced levels of biologically beneficial polyunsaturated PC species [29], whereas PSC is associated with increased levels of saturated PC species. The objective of this study was to quantify 21 serum PC species in healthy controls, patients with IBD, and patients with PSC to identify disease-specific PC signatures with potential as biomarkers for intestinal inflammation and PSC-associated liver fibrosis.

## 2. Materials and Methods

### 2.1. Patients and Controls

Patients receiving outpatient or inpatient care at a German university hospital between 12 June 2021 and 31 January 2025 were recruited for this study. Diagnoses of CD and UC were established using a combination of endoscopic findings, histopathology, and clinical presentation [30,31]. Fecal calprotectin, but not clinically or endoscopically assessed disease activity, was determined at the time of blood collection. Therefore, we used fecal calprotectin and CRP as measures for disease severity. PSC was diagnosed based on integrated clinical, biochemical, imaging, histological, and endoscopic assessments [32].

Exclusion criteria included the presence of coagulation disorders, pregnancy, or inability to provide informed consent. The control group comprised students, patients’ spouses, and hospital personnel. Written informed consent was obtained from all participants prior to inclusion in the study.

### 2.2. Measurement of PC Species

PC species were quantified by flow injection analysis Fourier-transform mass spectrometry (FIA-FTMS) using a hybrid quadrupole-Orbitrap mass spectrometer (Thermo Fisher Scientific, Waltham, MA, USA). The detailed methodology has been described previously [33]. PC 14:0/14:0 (1.29 nmol) and PC 22:0/22:0 (1.39 nmol) were used as internal standards and added before lipid extraction. Serum samples (10 µL) were extracted according to the procedure described by Bligh and Dyer [34]. Following phase separation, the lipid-containing chloroform layer was isolated using an automated pipetting system (Tecan Genesis RSP 150, Tecan Schweiz AG, Männedorf, Zürich, Switzerland) and subsequently dried under vacuum. The resulting residue was reconstituted in a chloroform-methanol-2-propanol (1:2:4, *v*/*v*/*v*) mixture supplemented with 7.5 mM ammonium formate. PC species were detected in negative ionization mode as formate adducts [M + HCOO]^−^ across an *m*/*z* window of 520–960. Instrument settings included a maximum injection time of 200 ms, an automated gain control target of 1 × 10^6^, acquisition of three microscans, and a resolving power of 140,000 at *m*/*z* 200. Quantitative analysis was achieved by scaling the known amount of the added internal standard by the ratio of the analyte signal to the internal standard signal.

The designation PC 32:1 indicates a phosphatidylcholine species comprising one saturated and one monounsaturated fatty acid with a total acyl chain length of 32 carbons; however, this method does not allow precise identification of the individual fatty acid constituents. For phosphatidylethanolamine quantification, PE 14:0/14:0 and PE 20:0/20:0 (di-phytanoyl) were used as internal standards, with additional methodological details provided in [35]. Analysis of cholesterol, triglycerides, and diacylglycerol has been described before [36].

### 2.3. Liver Stiffness Measurement

Liver stiffness was assessed using noninvasive acoustic radiation force impulse (ARFI) elastography. Measurements were taken from eight separate hepatic regions, and the median value was calculated to represent liver stiffness. Ultrasound imaging and data collection were performed using a Logiq™ E10 system (GE Healthcare, Munich, Germany) [37,38]. Liver stiffness data were obtained within six months of serum collection. Since the change in liver stiffness in patients with PSC/PSC-IBD within one year is small [39], this timeframe is considered appropriate.

### 2.4. Statistical Analysis

Results are reported as mean ± standard deviation. Normality of the distribution for individual PC species was evaluated using the Kolmogorov–Smirnov test, with *p*-values > 0.05 indicating consistency with normality. Group differences involving three or more cohorts were analyzed using one-way analysis of variance (ANOVA), followed by Bonferroni-corrected post hoc comparisons. For pairwise group comparisons, ANOVA test was applied, and *p*-values were adjusted by multiplying by 21 to account for the total number of analyzed PC species. Associations between variables were examined using Pearson’s correlation analysis, and *p*-values were adjusted by multiplying by 21 to account for the total number of analyzed PC species. Categorical variables were compared using the χ^2^ test. Diagnostic accuracy was evaluated through receiver operating characteristic (ROC) curve analysis. Multiple linear regression and binomial logistic regression analyses were also performed. All statistical procedures were performed using IBM SPSS Statistics (version 31.0; IBM Corp., Armonk, NY, USA). Statistical significance was defined as a two-sided *p*-value below 0.05.

## 3. Results

### 3.1. Study Cohorts

This study included 57 patients with inflammatory bowel disease (IBD), 26 female and 31 male patients (see Table 1 for specifics). Among these patients, 40 had Crohn’s disease (CD), and 17 had ulcerative colitis (UC). The study also included 20 patients with primary sclerosing cholangitis (PSC). Seven of these patients had isolated PSC, and 13 had PSC with underlying IBD. As healthy controls, eleven women and five men participated; the sex distribution of cases and controls did not differ significantly (*p* > 0.05). The average age of the control group, which was 52 years and ranged from 24 to 78 years, was higher than that of the patients with IBD (*p* < 0.05). Serum was collected only from controls with normal stature who reported good health. However, for the controls, body mass index (BMI) and clinical laboratory measurements were not recorded. Serum adiponectin levels (which decline with higher BMI [40]), soluble CD163 (a marker of macrophage activation which is increased in obesity [41]), and serum proprotein convertase subtilisin/kexin type 9 (which regulates serum cholesterol [42]), as well as total cholesterol and triglyceride levels of controls and patients with IBD, were similar (Table S1). Serum cholesterol of the controls was below 5.2 mmol/L, which is considered normal. Serum diacylglycerol of patients with IBD was higher compared to controls (Table S1). Although these markers, aside from cholesterol, are not standard laboratory measures, their associations with obesity, inflammation, and dyslipidemia are well described. Almost similar levels in patients with IBD and controls suggest that the two groups are not too different.

Serum levels of aminotransferases, gamma-glutamyltransferase, alkaline phosphatase, and bilirubin were higher in PSC than in IBD (Table 1).

### 3.2. Serum PC Species Levels of Patients and Controls

**Figure 1:**
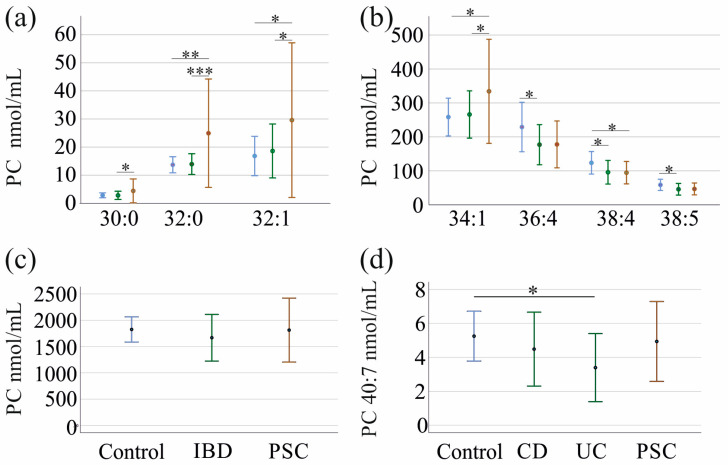
Serum PC species levels in patients with inflammatory bowel disease (IBD) and primary sclerosing cholangitis (PSC) are shown as mean ± standard deviation. Controls: blue bars, IBD: green bars, PSC: brown bars. (**a**) PC 30:0, 32:0, and 32:1 in the serum of controls and patient cohorts; (**b**) PC 34:1, 36:4, 38:4, and 38:5 in the serum of controls and patient cohorts; (**c**) Total PC levels in controls and patients; (**d**) PC 40:7 in controls, patients with Crohn’s disease (CD), ulcerative colitis (UC), and PSC Statistical test: one-way ANOVA followed by Bonferroni-corrected post hoc comparisons, * *p* < 0.05, ** *p* < 0.01, *** *p* < 0.001).

The serum levels of 21 PC species were measured in patients with IBD and PSC, as well as in healthy controls (Table 2). PC 30:0, 32:0, 32:1, and 34:1 were higher in patients with PSC than in those with IBD (Table 2 and Figure 1a,b). PC 32:0, 32:1, and 34:1 were elevated in PSC patients compared to controls. PC 36:4, 38:4, and 38:5 were lower in IBD patients than in controls. These species were also low in PSC, with this difference being significant for PC 38:4 (see Table 2 and Figure 1a,b). Total PC levels in these three groups were similar (Table 2 and Figure 1c).

Phosphatidylethanolamine (PE) 34:1 was higher in PSC than in healthy controls (*p* = 0.019). All other PE species (PE 32:1, 34:2, 34:3, 36:1, 36:2, 36:3, 36:4, 36:5, 38:1, 38:2, 38:3, 38:4, 38:5, 38:6, 40:4, 40:5, and 40:6) were similar in controls, patients with IBD, and patients with PSC (all *p* > 0.05).

Similar levels of all PC species were observed in patients with CD and UC (*p* > 0.05 for all). Serum PC 40:7 was lower in patients with UC than in controls (Figure 1d).

### 3.3. Serum PC Species of Patients with IBD in Relation to Inflammation

Patients with CD and UC had similar levels of all PC species, and correlations were analyzed in the entire cohort. In patients with IBD, PC 34:2, 36:2, 36:3, 36:4, 38:5, and total PC levels negatively correlated with C-reactive protein (CRP) and fecal calprotectin. In addition, PC 32:2, 34:3, 36:1, 38:3, 38:4, and 40:5 negatively correlated with fecal calprotectin, and PC 40:7 with CRP (Table 3).

The cut-off for the fecal calprotectin assay in the diagnosis of IBD is 120 µg/g. The 16 patients with fecal calprotectin > 120 µg/g had lower PC 36:2 (*p* = 0.014), 36:3 (*p* < 0.001), 36:4 (*p* = 0.001), 36:5 (*p* = 0.010), 38:3 (*p* = 0.003), 38:4 (*p* = 0.002), 38:5 (*p* < 0.001), 38:7 (*p* = 0.035), 40:5 (*p* = 0.016), 40:6 (*p* = 0.042) and total PC levels (*p* = 0.002) than the 41 patients with fecal calprotectin levels < 120 µg/g (Statistical test: ANOVA and multiplication by 21). The areas under the receiver operating characteristic curves (AUROCs) for all these PC species were <0.4, indicating they are not of diagnostic value.

There was no difference in the analyzed PE species between patients with calprotectin levels below or above the cutoff (all *p* > 0.05).

PC species that shared the same acyl chain length or the same degree of unsaturation were grouped together. PCs with 36 (*p* = 0.004), 38 (*p* = 0.004), and 40 (*p* = 0.013) acyl chains and three (*p* = 0.002), four (*p* = 0.006), five (*p* = 0.002), or six (*p* = 0.035) double bonds were lower in patients with fecal calprotectin > 120 µg/g compared to patients with levels below of this cut-off.

**Figure 2:**
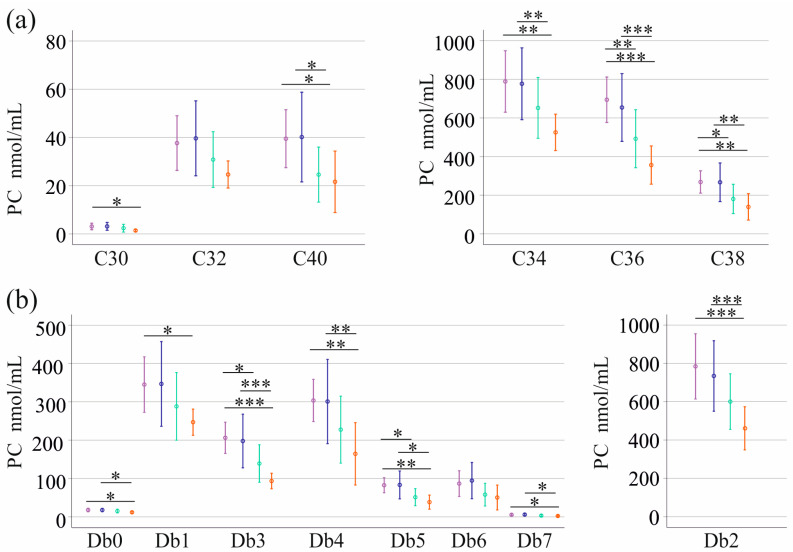
Serum PC species (median ± standard deviation) in relation to intestinal inflammation (calprotectin levels: <50 µg/g: pink bars, 50–<150 µg/g: blue bars, >150 and <500 µg/g: green bars, >500 µg/g: orange bars. (**a**) PC species grouped by identical acyl chain length. (**b**) PC species grouped by an identical number of double bonds (Db). * *p* < 0.05; * *p* < 0.01; *** *p* < 0.001. Statistical test: One-way ANOVA with post hoc Bonferroni.

Patients were also categorized by fecal calprotectin levels to assess associations between PC species and disease severity. All but PCs with C32 and PCs with six double bonds were reduced in patients with very high (>500 µg/g) calprotectin levels compared with those with very low (<50 µg/g) calprotectin levels (Figure 2). PC C34, C36, C38, and C40 were also reduced in patients with high calprotectin levels compared to those with levels between 50 and 150 µg/g. PC C36 and C38 also differed between those with low calprotectin levels and those with levels >150 and <500 µg/g (Figure 2). No significant association with fecal calprotectin levels was observed when PE species were grouped by identical acyl chain length or by the same number of double bonds. (all *p* > 0.05).

Patients with the highest calprotectin had increased PC34/total PC levels (% PC34) compared to those with low calprotectin (*p* = 0.003) and those with calprotectin levels between 50 and 150 µg/g (*p* = 0.019). PC36/total PC levels (% PC36) were lower in patients with high calprotectin than in those with low calprotectin (*p* < 0.001) and those with calprotectin >50 and <150 µg/g (*p* = 0.005).

PCs with 0, 1, 2, 3, 5, and 7 double bonds were reduced in patients with high calprotectin compared to patients with calprotectin levels <50 µg/g. PCs with 0, 2, 3, 4, 5, and 7 double bonds were also reduced in patients with high calprotectin compared to patients with calprotectin levels >50 and <150 µg/g. PCs with 3 or 5 double bonds differed between those with low calprotectin levels and those with levels >150 and <500 µg/g.

PCs with one double bond (PCDB1)/total PC levels (% PCDB1) were higher in patients with high calprotectin than in those with low calprotectin (*p* = 0.012). PCDB3/total PC levels (% PCDB3) were lower in patients with high calprotectin than in those with low calprotectin (*p* = 0.001) and those with calprotectin >50 and <150 µg/g (*p* = 0.011).

The PC/PE ratio was not associated with IBD severity (*p* = 0.097), and there was no difference in serum total PE levels between patients with low and high fecal calprotectin (*p* = 0.295), thereby ruling out the possibility that lower PC levels are associated with higher PE as a possible underlying mechanism.

Multiple linear regression analysis revealed that cholesterol (*p* < 0.001) and PE (*p* < 0.001), but not triglycerides (*p* = 0.115) or diacylglycerol (*p* = 0.089), predicted serum PC levels in IBD (F(4.51) = 71.064, *p* < 0.001).

### 3.4. Serum PC Species of Patients with PSC and Liver Disease Severity

In patients with PSC, none of the PC species showed a significant correlation with C-reactive protein or fecal calprotectin (all *p* > 0.05). Alanine aminotransferase levels were not associated with these PC levels. PC species with shorter acyl chains and fewer double bonds were positively correlated with aspartate aminotransferase, gamma-glutamyltransferase, alkaline phosphatase, bilirubin, and the MELD score. Total PC levels were positively correlated with gamma-glutamyltransferase and alkaline phosphatase (Table 4).

**Figure 3:**
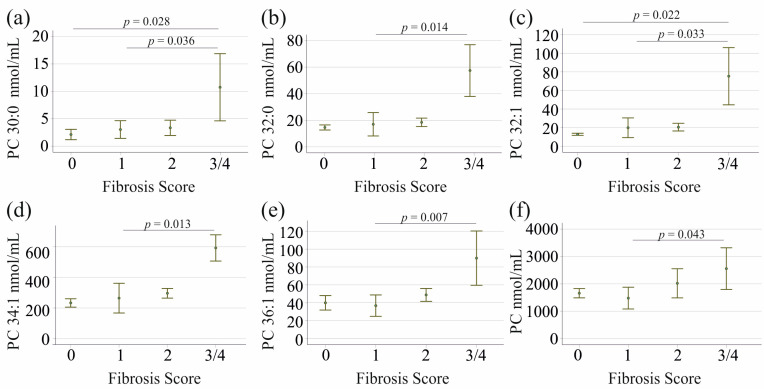
PC species levels (mean ± standard deviation) in relation to liver fibrosis. Liver fibrosis scores of 19 patients were documented (F0 = 3, F1 = 9, F2 = 3, F3 = 1, and F4 = 3), and patients with F3/F4 fibrosis, indicating advanced liver disease, were grouped together. (**a**) PC 30:0; (**b**) PC32:0; (**c**) PC 32:1; (**d**) PC34:1; (**e**) PC 36:1 and (**f**) total PC in patients with PSC stratified for liver fibrosis scores (F0: no fibrosis, F1: absent or mild fibrosis, F2: significant fibrosis, F3: severe fibrosis, F4: cirrhosis). Statistical Test: One-way ANOVA with post hoc Bonferroni.

PC 30:0, PC 32:0, PC 32:1, PC 34:1, and PC 36:1 of patients with advanced liver fibrosis were increased (Figure 3). Liver fibrosis scores of 19 patients were documented (F0 = 3, F1 = 9, F2 = 3, F3 = 1 and F4 = 3).

Total PC levels (*p* = 0.046) but not total PE levels (*p* = 0.280) were associated with fibrosis scores. The analysis of PE species did not reveal any changes across higher fibrosis stages (*p* > 0.05 for all).

The AUROC for discriminating PSC from IBD was highest for PC 32:0, at 0.687 ± 0.079 (*p* = 0.018). The AUROCs for all other PC species were lower and not significant. The AUROC for alkaline phosphatase was 0.809 ± 0.066 (*p* < 0.001), indicating that PC 32:0 does not provide additional diagnostic value.

Binomial logistic regression showed that alkaline phosphatase discriminated PSC from IBD (*p* < 0.001), with 98.1% specificity and 50% sensitivity. PC 32:0 with a specificity of 98.2% and a sensitivity of 35% (*p* < 0.001). The specificity was 96.2%, and the sensitivity was 50% when both were used. This shows that PC 32:0 is not suitable as a stand-alone or additional marker to alkaline phosphatase for PSC diagnosis.

PC 32:0 did not discriminate between the 13 PSC-IBD patients and IBD patients (*p* = 0.239).

Multiple linear regression showed that fibrosis stage, but not sex, predicts PC 30:0 (F(2,16) = 9.481, *p* = 0.002; sex *p* = 0.429, fibrosis *p* < 0.001), PC 32:0 (F(2,16) = 8.540, *p* = 0.003; sex *p* = 0.343, fibrosis *p* < 0.001), PC 32:1 (F(2,16) = 13.342, *p* < 0.001; sex *p* = 0.794, fibrosis *p* < 0.001), PC 34:1 (F(2,16) = 19.403, *p* < 0.001; sex *p* = 0.611; fibrosis *p* < 0.001) and PC 36:1 (F2,16) = 10.592, *p* < 0.001; sex *p* = 0.542; fibrosis *p* = 0.002).

Multiple linear regression analysis revealed that triglycerides (*p* = 0.012) and diacylglycerol (*p* = 0.016), but not cholesterol (*p* = 0.207) or PE (*p* = 0.782), predicted serum PC 32:0 levels in PSC (F(4,15) = 3.131, *p* = 0.046). Notably, none of these lipids changed with fibrosis stages (all *p* > 0.05).

### 3.5. Associations of PC Species with Age, Body Mass Index, Sex, Disease Duration, Current Medication, and Comorbidities

In patients with IBD or PSC, PC species did not correlate with age or BMI. PC 30:0 (*p* = 0.042), PC 32:1 (*p* = 0.021), PC 32:2 (*p* < 0.001), and PC 34:3 (*p* < 0.001) were all higher in female patients with IBD than in male patients. However, such a sex-specific difference was not observed in the control group or in patients with PSC, both of which were small cohorts.

Disease duration was documented for 7 patients with PSC (11.4 ± 15.6 years) and 38 patients with IBD (13.4 ± 11.7 years), but PC species did not correlate with time since first diagnosis (all *p* > 0.05).

Eighteen patients with PSC received ursodeoxycholic acid therapy, which precluded meaningful statistical evaluation. Among IBD patients, serum PC species concentrations did not differ substantially from those of patients treated with different drugs. This included 14 patients treated with corticosteroids, 14 receiving mesalazine, 15 undergoing anti–tumor necrosis factor therapy, and 13 treated with anti–IL-12/23 agents (all *p* > 0.05). However, these findings should be interpreted with caution, as most patients were receiving multiple concomitant medications, potentially limiting the reliability of the analysis.

Only two patients with IBD were on statin therapy, and one patient with PSC was on metformin; statistical analysis could not be performed.

Hypertension in seven patients with IBD was not related to altered PC species serum levels (*p* > 0.05).

## 4. Discussion

**Figure 4:**
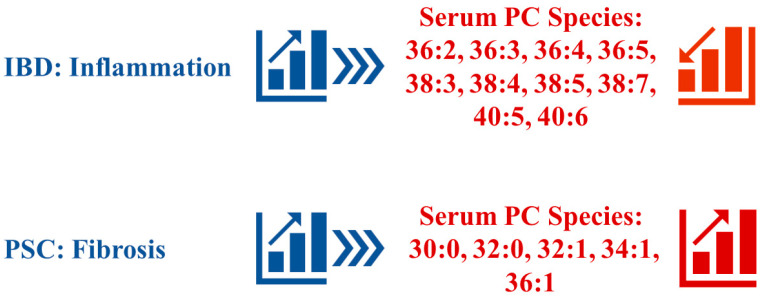
Reciprocal association of serum PC species with inflammation in IBD and fibrosis in PSC.

This study identifies reciprocal serum PC signatures in IBD and PSC. In patients with IBD, 11 of the 21 analyzed PC species were inversely associated with fecal calprotectin, whereas in PSC, five PC species correlated positively with the MELD score and were significantly elevated in individuals with advanced fibrosis. These findings suggest that intestinal inflammation is associated with a broad reduction in multiple PC species, largely independent of chain length or degree of unsaturation, whereas advanced liver fibrosis is associated with a more selective increase in shorter-chain saturated or monounsaturated PCs (Figure 4). Thus, rather than reflecting a uniform disturbance of PC metabolism, serum PC profiles appear to capture disease-specific processes across the gut–liver axis.

Serum levels of PC 30:0, 32:0, 32:1, and 34:1 were higher in patients with PSC than in those with IBD. All of these species showed positive correlations with fibrosis scores and laboratory markers of liver disease, supporting the interpretation that their elevation in PSC reflects advanced liver fibrosis. Among all measured species, PC 32:0 was the only one that significantly discriminated PSC from IBD. Importantly, PC 32:0 did not correlate with CRP or fecal calprotectin, arguing against underlying intestinal inflammation as a relevant confounder. PC 32:0 levels in patients with PSC were not predicted by cholesterol and PE levels, and only modest effects of triglycerides and diacylglycerols were observed. This shows that the increase in PC 32:0 in cirrhosis is not purely a marker of cirrhosis-associated dyslipidemia [43].

However, the AUROC of PC 32:0 was substantially lower than that of alkaline phosphatase; therefore, it is not a sufficiently valuable standalone diagnostic marker for PSC. Combining PC 32:0 with alkaline phosphatase levels is also not superior to alkaline phosphatase alone, showing that serum PC 32:0 is not a diagnostic biomarker for PSC. Notably, serum PC 32:0 has been identified as an excellent marker for the diagnosis of liver cirrhosis in patients with HCV [25]. Higher serum PC 32:0 levels in cirrhotic compared with non-cirrhotic, HBV-infected patients have also been reported [26]. Five of the analyzed PC species were significantly increased in patients with fibrosis scores of 3 or 4, suggesting high levels are also present in PSC-associated cirrhosis. As the PSC cohort was small and included patients with PSC and PSC-IBD, this association needs confirmation in larger cohorts.

Overall, the evidence suggests that elevated PC 32:0 levels are linked to liver cirrhosis and reduced hepatic function. In routine clinical practice, reliably diagnosing liver fibrosis without invasive procedures remains difficult. ARFI measurements are higher in patients with advanced fibrosis and correlate with inflammatory activity [44]. Similarly, the fibrosis-4 score is derived from aminotransferase values, which can also be increased in the context of inflammation [45]. In our cohort, serum PC 32:0 did not correlate with CRP or fecal calprotectin levels and remained unchanged in patients with HCV after viral elimination, which nearly normalizes systemic inflammation [25]. Therefore, serum PC 32:0 may represent an additional non-invasive marker of liver cirrhosis of different etiologies, with the potential advantage that its systemic levels are less affected by chronic inflammation. As our PSC cohort was small, confirmation in much larger cohorts is requested. However, PC species levels, either alone or in combination, are not appropriate for clinical use, such as the diagnosis of PSC, and do not seem suitable for monitoring the lower stages of fibrosis.

The levels of PC 36:4, 38:4, and 38:5 were lower in the serum of patients with IBD than in healthy controls. These species were negatively correlated with fecal calprotectin, indicating that their decline in IBD is linked to intestinal inflammatory activity. However, total serum PC levels in patients with IBD were similar to those in healthy controls, in accordance with the findings of Fan et al. [10]. Daniluk et al. reported decreased plasma PC levels in children with CD compared with healthy controls, whereas the reduction in children with UC was not statistically significant. In that study, which included 10 healthy controls, 10 children with UC, and 9 with CD, the CD group had high CRP levels, and the greater inflammatory burden may have contributed to the decline in PC levels [11].

Notably, whereas the PC species associated with fibrosis had shorter acyl chains and no or only one double bond, no such structural preference was observed for the PC species that declined during inflammation. Six PC species were negatively correlated with CRP, and eleven with fecal calprotectin. This suggests a broader reduction in circulating PC species during active intestinal inflammation, although not all changes were significant.

Impaired transport of PC across intestinal tight junctions has been implicated as a contributing factor in the development of UC. PC present in intestinal mucus is derived from circulating lipoproteins, and oral PC supplementation effectively improved inflammation in patients with UC [7]. Whether low serum PC levels in active IBD may contribute to intestinal PC depletion and further exacerbate colitis requires further study.

Patients with active IBD had higher %PC C34 and %PC DB1 levels and lower %PC C36 and %PC DB3 levels, indicating a modest shift towards PC species with fewer carbon atoms and fewer double bonds. Importantly, PC 30:0, 32:0, 32:1, and 34:1, which were increased in patients with liver fibrosis, did not decline in patients with IBD and were not correlated with markers of inflammation.

Lower serum PC levels in inflammation are likely attributable to reduced lipoprotein concentrations in patients with active IBD [46]. PC species show similar distributions in low-density lipoproteins (LDLs) and high-density lipoproteins (HDLs) [47], and both LDL and HDL are reduced in patients with active IBD [46]. PC levels in patients with IBD were indeed predicted by serum cholesterol. However, serum PE levels in our cohort were quite normal, and both of these lipid classes were associated with serum PC levels in IBD, with the underlying pathways still to be identified. PC synthesis is also influenced by inflammatory signaling, with pro-inflammatory cytokines modulating the expression and activity of enzymes involved in its biosynthetic pathways. Chronic inflammation may also increase PC turnover and degradation, thereby reducing PC availability in affected tissues [7,48].

Notably, serum levels of PE species did not change with inflammation in IBD or fibrosis in PSC, indicating that phospholipid levels are not generally altered in these conditions. Phospholipid synthesis occurs primarily in the endoplasmic reticulum [49], and in our observational study, there is no evidence that the activities of these enzymes are significantly impaired.

By contrast, the mechanisms underlying the selective increase in short-chain PCs in liver cirrhosis remain completely unclear. Patients with alcoholic liver cirrhosis have been reported to exhibit increased concentrations of saturated fatty acids, including palmitic acid, alongside decreased levels of polyunsaturated fatty acids in serum phospholipids when compared with healthy individuals [50]. That study suggested that malnutrition contributes to this unfavorable fatty acid profile in cirrhosis [50]. Although we identified higher levels of saturated and monounsaturated PCs in patients with PSC, we did not observe a decline in polyunsaturated PC species. Although body mass index was similar in patients with PSC and IBD, patients with PSC often report suboptimal dietary quality [51]. Malnutrition is also a concern in IBD [52], making it unlikely that altered PC levels are attributable solely to dietary factors. The nutritional status of our patients has not been documented. The reduced levels of serum PC species in patients with IBD, along with the higher levels of mostly short-chain fatty acid PCs in PSC, may initially suggest an altered dietary intake of these lipids. Because these alterations showed partial associations with inflammation or fibrosis scores, it is more plausible that they reflect the extent of disease severity rather than differences in diet.

Phosphatidylethanolamine N-methyltransferase (PEMT) converts PE to PC [4]. However, PE species were not associated with inflammation in IBD or fibrosis in PSC, arguing against altered PEMT activity as a major explanation for the observed findings. However, measuring only serum PC and PE levels is insufficient to definitively rule out a role for PEMT in the pathophysiology of IBD and PSC.

Stremmel et al. suggested that the paracellular transport of PC from the blood to the apical side of cholangiocytes is impaired in patients with PSC [20]. Biliary phospholipids are mostly PCs, with C34:1 and C34:2 species being among the most abundant [53]. These PC species correlated positively with routine measures of liver disease in our cohort, an observation that warrants confirmation in larger study groups. It can be hypothesized that higher levels of certain PC species in patients with PSC result from impaired biliary transport, a suggestion that requires experimental confirmation.

Previous studies have identified differences in plasma PC levels between patients with CD and UC. In our cohort, however, patients with CD and UC showed similar levels of all measured PC species. PC 40:7, which did not differ between controls and patients with CD, was significantly lower in patients with UC compared with controls, but did not differ between CD and UC. As this difference was small, this observation warrants confirmation in future studies.

In patients with IBD or PSC, PC species did not correlate with body mass index. A study addressing whether body levels of PCs and LPCs are reliable biomarkers of obesity concluded that the available data remain controversial [54]. PC 32:0 in blood has been shown to correlate with visceral fat mass in males [55], but whether a sex-specific association of systemic PC levels with body fat distribution exists requires further analysis.

In patients with HCV, age was positively associated with PC 30:0, PC 32:0, and PC 33:1 [25]. In healthy Japanese men, several PC species increased with age, and this effect was largely absent in females [56]. In our cohort, PC species were not correlated with age in patients or controls. Further studies are required to determine whether age influences serum PC levels.

PC 30:0, PC 32:1, PC 32:2, and PC 34:3 were higher in female than in male patients with IBD. In patients with HCV, sex-specific differences were not observed [25]. Except for PC 16:0/22:6, which was higher in the plasma of healthy females, other PC species showed no sex-specific distribution [57]. This suggests that sex may act as a confounding factor when analyzing circulating PC species in IBD. However, our sample size was too small to adjust robustly for these potential confounders. Sex, however, was not a contributing factor in the association between PC species and advanced fibrosis.

This study has several limitations. A limitation of this study is that BMI, laboratory measurements, nutritional status, and dietary lipid intake were unavailable for the control group, thereby restricting direct comparisons between patients and healthy controls. However, there was not sufficient serum left for further analysis. Moreover, samples from controls and patients have been analyzed in a single batch to obtain optimal results. Nevertheless, among individuals with IBD or PSC, PC species showed no significant association with age or BMI, suggesting that these factors are unlikely to have substantially influenced the findings. Importantly, this limitation does not affect the observed relationships between PC species and disease severity within the patient cohorts. The small size of the PSC cohort is a further limitation. Liver fibrosis was assessed using ARFI, and no additional non-invasive fibrosis scores were obtained. Finally, the specific fatty acids of the PC species could not be determined. The principal species contributing to PC 32:0 and PC 34:1 are PC 16:0/16:0 and PC 16:0/18:1, respectively, while other PC lipids display a comparatively uniform composition [15]. Thus, understanding the functional implications is constrained by the lack of comprehensive characterization of fatty acid side chains, with measurements limited to total carbon atoms and double-bond content. This monocentric study included only German residents; therefore, the findings may not be generalizable to other racial or ethnic populations.

## 5. Conclusions

In summary, this study highlights distinct and opposing relationships between circulating PC species and disease severity in IBD and PSC. Active IBD was associated with reduced concentrations of several PC species, particularly unsaturated forms, and these reductions correlated with the degree of intestinal inflammation. In contrast, progressive liver fibrosis in PSC was linked to elevated levels of shorter-chain saturated and monounsaturated PC species, a pattern that is consistent with observations reported in cirrhosis arising from various underlying causes.

The observations in the IBD cohort further support previous findings suggesting that diminished intestinal PC availability contributes to mucosal inflammation. They also reinforce the concept that increasing PC availability, reflected by higher circulating PC concentrations, may help mitigate inflammatory activity in the colon [7].

## Figures and Tables

**Table 1:** The patient cohort’s characteristics. The values that are displayed are the mean ± standard deviation. Because not all laboratory values were normally distributed, the statistical test used was the Mann–Whitney U test. The Chi-square test was used for categorical variables. Values were not corrected for multiple comparisons. * *p* < 0.05, ** *p* < 0.01, *** *p* < 0.001.

Characteristics	Inflammatory Bowel Disease	Primary Sclerosing Cholangitis
Number (female/male)	57 (26/31)	20 (8/12)
Age (years)	43 ± 14	45 ± 16
Body mass index (kg/m^2^)	25 ± 6	25 ± 8
C-reactive protein (mg/L)	10 ± 22	17 ± 28
Fecal calprotectin (µg/g)	211 ± 388	51 ± 64 *
Aspartate aminotransferase (U/L)	24 ± 7	60 ± 53 *
Alanine aminotransferase (U/L)	21 ± 11	46 ± 53
Gamma-glutamyltransferase (U/L)	30 ± 21	120 ± 136 **
Alkaline phosphatase (U/L)	68 ± 21	194 ± 160 ***
Bilirubin (mg/dL)	0.5 ± 0.4	3.3 ± 5.4 **
Model of end-stage liver disease score	Not defined	7.5 ± 2.7 (13 patients)
Cholesterol nmol/mL	4643 ± 1383	4322 ± 1436
Triglycerides nmol/mL	1400 ± 806	1731 ± 1022
Diacylglycerol nmol/mL	35 ± 22	39 ± 23
Type 2 diabetes	1	1
Hypertension	7	0

**Table 2:** Mean ± standard deviation of serum PC levels of patients and controls. Statistical test: One-way ANOVA with post hoc Bonferroni. *, ^%^, ^&^ *p* < 0.05, ^%%^ *p* < 0.01, *** *p* < 0.001. Identical symbols were used to indicate significant differences between cohorts.

PC nmol/mL	Control (N = 16)	IBD (N = 57)	PSC (N = 20)
30:0	2.83 ± 0.89	2.80 ± 1.48 *	4.41 ± 4.26 *
32:0	13.70 ± 2.87 ^%%^	13.92 ± 3.70 ***	24.93 ± 19.30 *** ^%%^
32:1	16.83 ± 7.01 ^%^	18.61 ± 9.59 *	29.59 ± 27.52 *^%^
32:2	3.33 ± 1.12	3.25 ± 1.80	3.43 ± 1.77
34:1	258.45 ± 55.55 ^%^	266.01 ± 69.55 *	334.19 ± 153.33 *^%^
34:2	466.82 ± 74.17	456.33 ± 124.08	480.18 ± 173.71
34:3	15.65 ± 4.25	13.99 ± 5.78	15.83 ± 8.60
36:1	41.11 ± 11.39	41.88 ± 14.00	49.22 ± 26.04
36:2	263.70 ± 52.83	248.53 ± 76.83	262.11 ± 106.63
36:3	143.94 ± 19.99	129.77 ± 43.00	136.34 ± 54.32
36:4	229.05 ± 72.77 ^&^	177.16 ± 59.10 ^&^	177.89 ± 68.91
36:5	25.73 ± 17.78	19.29 ± 11.66	16.00 ± 11.87
38:3	41.98 ± 14.48	38.00 ± 17.02	36.05 ± 14.76
38:4	123.72 ± 33.28 ^&%^	95.96 ± 34.86 ^&^	94.62 ± 32.96 ^%^
38:5	58.69 ± 16.62 ^&^	45.84 ± 17.17 ^&^	46.77 ± 17.49
38:6	76.04 ± 28.93	60.93 ± 30.13	60.96 ± 29.90
38:7	1.04 ± 0.58	0.67 ± 0.67	0.67 ± 0.85
40:4	3.73 ± 1.31	3.22 ± 1.25	3.33 ± 1.28
40:5	10.11 ± 3.23	8.31 ± 3.57	9.55 ± 3.87
40:6	23.83 ± 7.30	19.97 ± 10.25	22.08 ± 12.60
40:7	5.25 ± 1.47	4.16 ± 2.17	4.94 ± 2.36
Total PC	1825.54 ± 238.93	1668.61 ± 443.70	1813.06 ± 609.08

**Table 3:** Correlation coefficients between PC species, C-reactive protein, and fecal calprotectin in patients with IBD. Statistical tests: Pearson’s correlation (*p*-values were corrected for multiple comparisons by multiplication by 21). * *p* < 0.05, ** *p* < 0.01, *** *p* < 0.001.

PC nmol/mL	C-Reactive Protein	Calprotectin
30:0	−0.223	−0.359
32:0	−0.275	−0.374
32:1	−0.178	−0.248
32:2	−0.315	−0.427 *
34:1	−0.309	−0.330
34:2	−0.407 *	−0.462 **
34:3	−0.397	−0.453 **
36:1	−0.337	−0.428 *
36:2	−0.414 *	−0.508 **
36:3	−0.485 **	−0.577 ***
36:4	−0.460 *	−0.507 **
36:5	−0.390	−0.356
38:3	−0.322	−0.473 **
38:4	−0.392	−0.451 **
38:5	−0.493 **	−0.508 **
38:6	−0.365	−0.303
38:7	−0.327	−0.310
40:4	−0.372	−0.380
40:5	−0.386	−0.409 *
40:6	−0.300	−0.295
40:7	−0.426 *	−0.378
Total PC	−0.470 **	−0.540 ***

**Table 4:** Pearson correlation coefficients were calculated to assess associations between PC species and standard laboratory markers of liver disease severity in patients with PSC. *p*-values were adjusted using a Bonferroni-type correction by multiplying them by 21, corresponding to the total number of PC species evaluated. ALT: Alanine aminotransferase, AP: alkaline phosphatase, AST: Aspartate aminotransferase, Gamma-GT: gamma-glutamyltransferase, MELD: model for end-stage liver disease. * *p* < 0.05, ** *p* < 0.01, *** *p* < 0.001.

PC nmol/mL	ALT	AST	Gamma-GT	AP	Bilirubin	MELD
30:0	0.472	0.780 **	0.629	0.791 **	0.643 *	0.879 **
32:0	0.498	0.828 ***	0.717 **	0.854 ***	0.782 **	0.936 ***
32:1	0.462	0.785 **	0.661 *	0.794 **	0.761 **	0.947 **
32:2	0.589	0.619	0.712 **	0.729 **	0.374	0.555
34:1	0.580	0.803 ***	0.801 ***	0.863 ***	0.742 **	0.920 ***
34:2	0.471	0.522	0.718 **	0.637	0.423	0.211
34:3	0.634	0.610	0.798 **	0.747 **	0.339	0.505
36:1	0.295	0.663 *	0.555	0.717 **	0.725 **	0.849 **
36:2	0.261	0.470 *	0.562	0.577	0.503	0.112
36:3	0.519	0.417	0.664 *	0.534 *	0.107	0.318
36:4	0.534	0.196	0.527	0.171	−0.242	0.205
36:5	0.494	0.230	0.536	0.423	−0.153	0.297
38:3	0.169	0.166	0.395	0.238	0.029	0.125
38:4	0.257	0.139	0.416	0.121	−0.100	0.073
38:5	0.365	0.094	0.393	0.182	−0.285	0.202
38:6	0.298	0.155	0.359	0.309	−0.148	0.203
38:7	0.511	0.309	0.548	0.508	−0.043	0.411
40:4	0.079	0.077	0.180	0.104	−0.094	0.283
40:5	0.134	0.127	0.241	0.190	−0.062	0.180
40:6	0.165	0.257	0.302	0.367	0.100	0.289
40:7	0.362	0.310	0.506	0.509 *	0.025	0.453
Total PC	0.555	0.633	0.800 ***	0.732 ***	0.457	0.479

## Data Availability

The raw data supporting the conclusions of this article will be made available by the authors on request.

## Supplementary Materials

The following supporting information can be downloaded at: https://www.mdpi.com/article/10.3390/biomedicines14071485/s1, Table S1: Levels of different proteins, cholesterol, triglycerides and diacylglycerol in serum of patients with IBD and controls. * *p* < 0.05.

## Abbreviations

The following abbreviations are used in this manuscript:

ALT	Alanine aminotransferase
AP	Alkaline phosphatase
AST	Aspartate aminotransferase
CD	Crohn’s Disease
GGT	Gamma-glutamyltransferase
HCV	Hepatitis C virus
IBD	Inflammatory Bowel Disease
PC	Phosphatidylcholine
PSC	Primary sclerosing cholangitis
UC	Ulcerative Colitis

## References

[1] Becker H.E.F., Demers K., Derijks L.J.J., Jonkers D., Penders J. (2023). Current evidence and clinical relevance of drug-microbiota interactions in inflammatory bowel disease. Front. Microbiol..

[2] Brown S.J., Mayer L. (2007). The immune response in inflammatory bowel disease. Am. J. Gastroenterol..

[3] de Souza H.S., Fiocchi C. (2016). Immunopathogenesis of IBD: Current state of the art. Nat. Rev. Gastroenterol. Hepatol..

[4] van der Veen J.N., Kennelly J.P., Wan S., Vance J.E., Vance D.E., Jacobs R.L. (2017). The critical role of phosphatidylcholine and phosphatidylethanolamine metabolism in health and disease. Biochim. Biophys. Acta Biomembr..

[5] Wang B., Tontonoz P. (2019). Phospholipid Remodeling in Physiology and Disease. Annu. Rev. Physiol..

[6] Barrios J.M., Lichtenberger L.M. (2000). Role of biliary phosphatidylcholine in bile acid protection and NSAID injury of the ileal mucosa in rats. Gastroenterology.

[7] Stremmel W., Weiskirchen R. (2024). Phosphatidylcholine in Intestinal Mucus Protects against Mucosal Invasion of Microbiota and Consequent Inflammation. Livers.

[8] Jacobsson L., Lindgarde F., Manthorpe R., Akesson B. (1990). Correlation of fatty acid composition of adipose tissue lipids and serum phosphatidylcholine and serum concentrations of micronutrients with disease duration in rheumatoid arthritis. Ann. Rheum. Dis..

[9] Raouf J., Idborg H., Englund P., Alexanderson H., Dastmalchi M., Jakobsson P.J., Lundberg I.E., Korotkova M. (2018). Targeted lipidomics analysis identified altered serum lipid profiles in patients with polymyositis and dermatomyositis. Arthritis Res. Ther..

[10] Fan F., Mundra P.A., Fang L., Galvin A., Moore X.L., Weir J.M., Wong G., White D.A., Chin-Dusting J., Sparrow M.P. (2015). Lipidomic Profiling in Inflammatory Bowel Disease: Comparison Between Ulcerative Colitis and Crohn’s Disease. Inflamm. Bowel Dis..

[11] Daniluk U., Daniluk J., Kucharski R., Kowalczyk T., Pietrowska K., Samczuk P., Filimoniuk A., Kretowski A., Lebensztejn D., Ciborowski M. (2019). Untargeted Metabolomics and Inflammatory Markers Profiling in Children With Crohn’s Disease and Ulcerative Colitis—A Preliminary Study. Inflamm. Bowel Dis..

[12] Iwatani S., Iijima H., Otake Y., Amano T., Tani M., Yoshihara T., Tashiro T., Tsujii Y., Inoue T., Hayashi Y. (2020). Novel mass spectrometry-based comprehensive lipidomic analysis of plasma from patients with inflammatory bowel disease. J. Gastroenterol. Hepatol..

[13] Tews H.C., Huss M., Elger T., Liebisch G., Horing M., Loibl J., Kandulski A., Muller M., Buechler C. (2026). Serum phosphatidylinositol depletion associates with fecal calprotectin and disease severity in female and male IBD patients. Lipids Health Dis..

[14] Ding N.S., McDonald J.A.K., Perdones-Montero A., Rees D.N., Adegbola S.O., Misra R., Hendy P., Penez L., Marchesi J.R., Holmes E. (2020). Metabonomics and the Gut Microbiome Associated With Primary Response to Anti-TNF Therapy in Crohn’s Disease. J. Crohns Colitis.

[15] Quell J.D., Romisch-Margl W., Haid M., Krumsiek J., Skurk T., Halama A., Stephan N., Adamski J., Hauner H., Mook-Kanamori D. (2019). Characterization of Bulk Phosphatidylcholine Compositions in Human Plasma Using Side-Chain Resolving Lipidomics. Metabolites.

[16] Lei H., Jiang Y., Chen Z., Yao J., Ma W., Huang Y., Zhang P., Xie Z., Zhu L., Tang W. (2025). Unveiling the influence of lipidomes on inflammatory bowel disease: A bidirectional mendelian randomization study. BMC Gastroenterol..

[17] Floreani A., De Martin S. (2021). Treatment of primary sclerosing cholangitis. Dig. Liver Dis..

[18] Kim Y.S., Hurley E.H., Park Y., Ko S. (2023). Primary sclerosing cholangitis (PSC) and inflammatory bowel disease (IBD): A condition exemplifying the crosstalk of the gut-liver axis. Exp. Mol. Med..

[19] van Munster K.N., Bergquist A., Ponsioen C.Y. (2023). Inflammatory bowel disease and primary sclerosing cholangitis: One disease or two?. J. Hepatol..

[20] Stremmel W., Lukasova M., Weiskirchen R. (2021). The neglected biliary mucus and its phosphatidylcholine content: A putative player in pathogenesis of primary cholangitis-a narrative review article. Ann. Transl. Med..

[21] Gauss A., Ehehalt R., Lehmann W.D., Erben G., Weiss K.H., Schaefer Y., Kloeters-Plachky P., Stiehl A., Stremmel W., Sauer P. (2013). Biliary phosphatidylcholine and lysophosphatidylcholine profiles in sclerosing cholangitis. World J. Gastroenterol..

[22] Mohajeri S., Bezabeh T., Ijare O.B., King S.B., Thomas M.A., Minuk G., Lipschitz J., Kirkpatrick I., Micflikier A.B., Summers R. (2019). In vivo (1) H MRS of human gallbladder bile in understanding the pathophysiology of primary sclerosing cholangitis (PSC): Immune-mediated disease versus bile acid-induced injury. NMR Biomed..

[23] Fan Z.K., Ma W.J., Zhang W., Li H., Zhai J., Zhao T., Guo X.F., Sinclair A.J., Li D. (2022). Elevated serum phosphatidylcholine (16:1/22:6) levels promoted by fish oil and vitamin D(3) are highly correlated with biomarkers of non-alcoholic fatty liver disease in Chinese subjects. Food Funct..

[24] Mazzini F.N., Cook F., Gounarides J., Marciano S., Haddad L., Tamaroff A.J., Casciato P., Narvaez A., Mascardi M.F., Anders M. (2021). Plasma and stool metabolomics to identify microbiota derived-biomarkers of metabolic dysfunction-associated fatty liver disease: Effect of PNPLA3 genotype. Metabolomics.

[25] Weigand K., Peschel G., Grimm J., Horing M., Krautbauer S., Liebisch G., Muller M., Buechler C. (2024). Serum Phosphatidylcholine Species 32:0 as a Biomarker for Liver Cirrhosis Pre- and Post-Hepatitis C Virus Clearance. Int. J. Mol. Sci..

[26] Chen S., Yin P., Zhao X., Xing W., Hu C., Zhou L., Xu G. (2013). Serum lipid profiling of patients with chronic hepatitis B, cirrhosis, and hepatocellular carcinoma by ultra fast LC/IT-TOF MS. Electrophoresis.

[27] Meikle P.J., Mundra P.A., Wong G., Rahman K., Huynh K., Barlow C.K., Duly A.M., Haber P.S., Whitfield J.B., Seth D. (2015). Circulating Lipids Are Associated with Alcoholic Liver Cirrhosis and Represent Potential Biomarkers for Risk Assessment. PLoS ONE.

[28] Virseda-Berdices A., Rojo D., Martinez I., Berenguer J., Gonzalez-Garcia J., Brochado-Kith O., Fernandez-Rodriguez A., Diez C., Hontanon V., Perez-Latorre L. (2022). Metabolomic changes after DAAs therapy are related to the improvement of cirrhosis and inflammation in HIV/HCV-coinfected patients. Biomed. Pharmacother..

[29] Yan D., Ye S., He Y., Wang S., Xiao Y., Xiang X., Deng M., Luo W., Chen X., Wang X. (2023). Fatty acids and lipid mediators in inflammatory bowel disease: From mechanism to treatment. Front. Immunol..

[30] Kucharzik T., Dignass A., Siegmund B. (2019). Aktualisierung der S3-Leitlinie Colitis ulcerosa 2019. Z. Gastroenterol..

[31] Sturm A., Maaser C., Calabrese E., Annese V., Fiorino G., Kucharzik T., Vavricka S.R., Verstockt B., van Rheenen P., Tolan D. (2019). ECCO-ESGAR Guideline for Diagnostic Assessment in IBD Part 2: IBD scores and general principles and technical aspects. J. Crohns Colitis.

[32] European Association for the Study of the Liver (2022). EASL Clinical Practice Guidelines on sclerosing cholangitis. J. Hepatol..

[33] Horing M., Ejsing C.S., Krautbauer S., Ertl V.M., Burkhardt R., Liebisch G. (2021). Accurate quantification of lipid species affected by isobaric overlap in Fourier-Transform mass spectrometry. J. Lipid Res..

[34] Bligh E.G., Dyer W.J. (1959). A rapid method of total lipid extraction and purification. Can. J. Biochem. Physiol..

[35] Dawczynski C., Plagge J., Jahreis G., Liebisch G., Horing M., Seeliger C., Ecker J. (2022). Dietary PUFA Preferably Modify Ethanolamine-Containing Glycerophospholipids of the Human Plasma Lipidome. Nutrients.

[36] Haberl E.M., Pohl R., Rein-Fischboeck L., Höring M., Krautbauer S., Liebisch G., Buechler C. (2021). Accumulation of cholesterol, triglycerides and ceramides in hepatocellular carcinomas of diethylnitrosamine injected mice. Lipids Health Dis..

[37] Goertz R.S., GaBmann L., Strobel D., Wildner D., Schellhaas B., Neurath M.F., Pfeifer L. (2019). Acoustic Radiation Force Impulse (ARFI) Elastography in Autoimmune and Cholestatic Liver Diseases. Ann. Hepatol..

[38] Sporea I., Bota S., Sirli R., Popescu A., Danila M., Jurchis A., Gradinaru-Tascau O., Martie A. (2013). The usefulnes of Acoustic Radiation Force Impulse (ARFI) Elastography (ARFI) for evaluation of liver fibrosis–large monocentric experience. Ultraschall Med..

[39] Fossdal G., Mjelle A.B., Wiencke K., Bjork I., Gilja O.H., Folseraas T., Karlsen T.H., Rosenberg W., Giil L.M., Vesterhus M. (2021). Fluctuating biomarkers in primary sclerosing cholangitis: A longitudinal comparison of alkaline phosphatase, liver stiffness, and ELF. JHEP Rep..

[40] Ismaiel A., Ciornolutchii V., Herrera T.E., Ismaiel M., Leucuta D.C., Popa S.L., Dumitrascu D.L. (2025). Adiponectin as a biomarker in liver cirrhosis-A systematic review and meta-analysis. Eur. J. Clin. Investig..

[41] Fjeldborg K., Christiansen T., Bennetzen M., Møller H.J., Pedersen S.B., Richelsen B. (2013). The macrophage-specific serum marker, soluble CD163, is increased in obesity and reduced after dietary-induced weight loss. Obesity.

[42] Bao X., Liang Y., Chang H., Cai T., Feng B., Gordon K., Zhu Y., Shi H., He Y., Xie L. (2024). Targeting proprotein convertase subtilisin/kexin type 9 (PCSK9): From bench to bedside. Signal Transduct. Target. Ther..

[43] Buechler C., Aslanidis C. (2020). Role of lipids in pathophysiology, diagnosis and therapy of hepatocellular carcinoma. Biochim. Biophys. Acta Mol. Cell Biol. Lipids.

[44] Peschel G., Grimm J., Buechler C., Gunckel M., Pollinger K., Aschenbrenner E., Kammerer S., Jung E.M., Haimerl M., Werner J. (2021). Liver stiffness assessed by shear-wave elastography declines in parallel with immunoregulatory proteins in patients with chronic HCV infection during DAA therapy. Clin. Hemorheol. Microcirc..

[45] Alem S.A., Abdellatif Z., Mabrouk M., Zayed N., Elsharkawy A., Khairy M., Musa S., Anwar I., Yosry A. (2019). Diagnostic accuracy of acoustic radiation force impulse elastography (ARFI) in comparison to other non-invasive modalities in staging of liver fibrosis in chronic HCV patients: Single-center experience. Abdom. Radiol..

[46] Chen H., Li W., Hu J., Xu F., Lu Y., Zhu L., Shen H. (2023). Association of serum lipids with inflammatory bowel disease: A systematic review and meta-analysis. Front. Med..

[47] Wiesner P., Leidl K., Boettcher A., Schmitz G., Liebisch G. (2009). Lipid profiling of FPLC-separated lipoprotein fractions by electrospray ionization tandem mass spectrometry. J. Lipid Res..

[48] Maia C., Fung C.W., Sanchez-Lopez E. (2025). Choline in immunity: A key regulator of immune cell activation and function. Front. Immunol..

[49] Gibellini F., Smith T.K. (2010). The Kennedy pathway—De novo synthesis of phosphatidylethanolamine and phosphatidylcholine. IUBMB Life.

[50] Ristic-Medic D., Takic M., Vucic V., Kandic D., Kostic N., Glibetic M. (2013). Abnormalities in the serum phospholipids fatty acid profile in patients with alcoholic liver cirrhosis-a pilot study. J. Clin. Biochem. Nutr..

[51] Lindqvist C., Ingre M., Kechagias S., Nilsson E., Molinaro A., Rorsman F., Bergquist A. (2025). Dietary Habits of Individuals With Primary Sclerosing Cholangitis-Poor Fat-Soluble Vitamin Intake and Dietary Quality. Liver Int..

[52] Scaldaferri F., Pizzoferrato M., Lopetuso L.R., Musca T., Ingravalle F., Sicignano L.L., Mentella M., Miggiano G., Mele M.C., Gaetani E. (2017). Nutrition and IBD: Malnutrition and/or Sarcopenia? A Practical Guide. Gastroenterol. Res. Pract..

[53] Hay D.W., Cahalane M.J., Timofeyeva N., Carey M.C. (1993). Molecular-Species of Lecithins in Human Gallbladder Bile. J. Lipid Res..

[54] Bellot P., Moia M.N., Reis B.Z., Pedrosa L.F.C., Tasic L., Barbosa F., Sena-Evangelista K.C.M. (2023). Are Phosphatidylcholine and Lysophosphatidylcholine Body Levels Potentially Reliable Biomarkers in Obesity? A Review of Human Studies. Mol. Nutr. Food Res..

[55] Szymanska E., Bouwman J., Strassburg K., Vervoort J., Kangas A.J., Soininen P., Ala-Korpela M., Westerhuis J., van Duynhoven J.P., Mela D.J. (2012). Gender-dependent associations of metabolite profiles and body fat distribution in a healthy population with central obesity: Towards metabolomics diagnostics. Omics.

[56] Kawanishi N., Kato Y., Yokozeki K., Sawada S., Sakurai R., Fujiwara Y., Shinkai S., Goda N., Suzuki K. (2018). Effects of aging on serum levels of lipid molecular species as determined by lipidomics analysis in Japanese men and women. Lipids Health Dis..

[57] West A.L., Michaelson L.V., Miles E.A., Haslam R.P., Lillycrop K.A., Georgescu R., Han L., Napier J.A., Calder P.C., Burdge G.C. (2021). Lipidomic Analysis of Plasma from Healthy Men and Women Shows Phospholipid Class and Molecular Species Differences between Sexes. Lipids.

