# Supplementary material for: Reciprocal Serum Phosphatidylcholine Signatures Are Related to Intestinal Inflammation in Inflammatory Bowel Disease and Liver Fibrosis in Primary Sclerosing Cholangitis—An Exploratory Study
Source: Biomedicines. 2026 Jun 30;14(7):1485. doi: 10.3390/biomedicines14071485 (PMC13404610; doi:10.3390/biomedicines14071485)
Supplement: Supplementary file 1 [file biomedicines-14-01485-s001.zip › biomedicines-4304650-supplementary.pdf]

Table S1: Levels of different proteins, cholesterol, triglycerides and diacylglycerol in serum of patients with IBD and controls. * $p < 0.05$.

Characteristics	IBD	Controls
Number (female/male)	57 (26/31)	16 (11/5)
Adiponectin (µg/ml)	3.9 ± 2.4	3.1 ± 1.8
sCD163 (ng/ml)	466 ± 210	567 ± 163
Cholesterol nmol/ml	4643 ± 1383	4662 ± 867
Triglycerides nmol/ml	1400 ± 806	1076 ± 479
Diacylglycerol nmol/ml	35 ± 22	23 ± 10 *
PSCK9 ng/ml	347 ± 222	316 ± 199
